# Pentraxin-3 as a Marker of Advanced Atherosclerosis Results from the Bruneck, ARMY and ARFY Studies

**DOI:** 10.1371/journal.pone.0031474

**Published:** 2012-02-03

**Authors:** Michael Knoflach, Stefan Kiechl, Alberto Mantovani, Ivan Cuccovillo, Barbara Bottazzi, Qingbo Xu, Qingzhong Xiao, Arno Gasperi, Agnes Mayr, Marlene Kehrer, Johann Willeit, Georg Wick

**Affiliations:** 1 Department of Neurology, Medical University, Innsbruck, Austria; 2 Istituto Clinico Humanitas IRCCS, Rozzano, Milan, Italy; 3 Department of Translational Medicine, University of Milan, Milan, Italy; 4 Fondazione Humanitas per la Ricerca, Rozzano, Milan, Italy; 5 Cardiovascular Division, BHF Centre, King's College London, London, United Kingdom; 6 Department of Neurology, Bruneck Hospital, Bruneck, Italy; 7 Department of Laboratory Medicine, Bruneck Hospital, Bruneck, Italy; 8 Department of Internal Medicine, Bruneck Hospital, Bruneck, Italy; 9 Laboratory of Autoimmunity, Biocenter, Innsbruck, Austria; Heart Center Munich, Germany

## Abstract

**Objective:**

Pentraxins like C-reactive protein are key components of the innate immune system. Recently, pentraxin-3 (PTX3) has been proposed to be a specific marker of vascular inflammation, yet its association with atherosclerosis is still unclear.

**Methods and Results:**

PTX3 serum levels were measured in three independent studies of 132 young men (ARMY Study), 205 young women (ARFY Study) and 562 individuals 55 to 94 years old (Bruneck Study). In contrast to C-reactive protein, PTX3 showed little relationships with classic vascular risk factors and pro-inflammatory conditions. In the population based Bruneck Study, PTX3 level was independently associated with prevalent cardiovascular diseases (multivariable odds ratio [95%CI] 3.09 [1.65–5.79]; P<0.001). Moreover, PTX3 level correlated with the severity of carotid and femoral atherosclerosis and was highest in individuals with multiple vascular territories affected. In contrast, there was no association with elevated intima-media thickness, a precursor lesion of atherosclerosis, in any of the three populations investigated.

**Conclusions:**

Level of PTX3 is independently associated with atherosclerosis and manifest cardiovascular disease but not early vessel pathology. Unlike C-reactive protein, PTX3 is not a component of the classic acute phase response (systemic inflammation) but appears to be more specific for vascular inflammation.

## Introduction

It is well documented, that immunological and inflammatory processes play a fundamental role in atherogenesis.[1]–[3] Pentraxins are key components of the humoral arm of the innate immune system.[4] The best known member of this protein family is C-reactive protein (CRP), a short pentraxin. Recently a long pentraxin – pentraxin-3 (PTX3) – has moved into the focus of research. It is expressed in monocytes, macrophages, endothelical cells, dentritic cells, fibroblasts and epithelial cells [4] – all of which are present in the vascular wall and increased in atherosclerotic plaques – and therefore a potential specific marker of inflammation and atherosclerotic changes of the vascular wall.[5]–[7] High PTX3 levels have been associated with unstable angina [8], adverse outcome after myocardial infarction [9] and heart failure [10]. In a subsample of the Cardiovascular Health Study, baseline PTX3 was associated with cardiovascular and all cause mortality, yet not with incident angina or myocardial infarction [11]. In contrast to CRP, which has a recent evolutionary history, PTX3 is structurally highly conserved from mice to men.[4] It shows little correlations with standard vascular risk factors [11], is not produced in the liver but locally in atherosclerotic lesions themselves [12] and was reported to accumulate during atherosclerosis progression in a murine model [13] - making PTX3 a promising and more specific marker for vascular inflammation.

The current study is the first to explore the association of PTX3 level with *in vivo* measurements of atherosclerosis in three independent population studies.

## Methods

### Ethics Statement

All participants gave a written informed consent, and each study was approved by the corresponding ethics committees (Innsbruck, Austria for the ARMY and ARFY studies and Bolzano, Italy for the Bruneck study).

### Study Design

The Bruneck study is a prospective population-based survey of the epidemiology and pathogenesis of atherosclerosis. At the 1990 baseline evaluation, the study population was recruited as a sex- and age-stratified random sample of all inhabitants of Bruneck (Bolzano Province, Italy) 40–79 years old (125 women and 125 men in the fifth to eighth decades each; n = 1000). A total of 93.6% participated, with the original data assessment completed for 919 subjects. The present investigation focused on the 2005 re-evaluation and involved 562 study participants aged 55–94 years (all Caucasians). Based on the questionnaires and logistics of the Bruneck Study two cross-sectional studies of young men and women were designed:

The **A**therosclerosis **R**isk Factors in **M**ale **Y**oungsters (ARMY) Study is a cross-sectional evaluation of young men performed in 2001. In Austria, every male citizen undergoes a thorough physical examination by experienced medical personnel to assess physical fitness for recruitment into the Austrian army in the year he turns 18 - except for those suffering from chronic diseases (e.g. diabetes) or permanent disabilities (<1.5%). In 132 of the initially participating 141 individuals sufficient amounts of serums samples were available (stored at −80°C) for measurement of PTX3.

The **A**therosclerosis **R**isk Factor in **F**emale **Y**oungsters (ARFY) Study is a cross-sectional ultrasound-based evaluation of risk factors for early vessel pathology in young women. Between April and June 2005, all female students of the Educational Centre West for Allied Health Professions (Innsbruck, Austria), 18 to 22 years old, were invited to participate. A total of 211 women, all upcoming healthcare professionals (nurses, medical technicians, physiotherapists, occupational therapists, logopedics, and dieticians) accepted the invitation. All participants were white and none had a history of cardiovascular disease. Data assessment was complete in 205 women, who formed the current study population. The three studies were similar in design, logistics and methodology. Details have been published before.[14]–[17]

### Clinical history and examination

The participants of the three studies underwent a thorough clinical examination and completed the same standardized questionnaires on current and past exposure to vascular risk factors. Waist was measured at the narrowest point between the costal margin and the iliac crest (on the naked abdomen). Body mass index (BMI) was calculated as body weight divided by the square of the height (in meters). The average number of cigarettes smoked per day was noted for each smoker and ex-smoker. Subjects were categorized as “smokers” if they reported regular consumption of >1 cigarette per week and lifetime consumption exceeded 40 cigarettes. Cigarette pack-years were calculated by multiplying the years of smoking by the packs smoked per day. Subjects were instructed to indicate their customary alcohol consumption during a typical day (in the case of regular alcohol consumption) or week (all alcoholic beverages consumed on weekdays and the weekend). Average alcohol intake was expressed as grams of ethanol per day and calculated from responses concerning the various types and amounts of alcoholic beverages consumed. Office blood pressure readings were taken after at least 10 minutes of rest and calculated as a mean of three independent readings. Hypertension was defined as blood pressure ≥140/90 mmHg or use of antihypertensive drugs. Social status was deduced from occupation of the household member with the highest income.[15] The sport index was calculated according to the Baecke formula.[18] Diabetes mellitus was diagnosed if the subject was being treated with insulin or oral hypoglycemic drugs, if the subject's fasting plasma level of glucose exceeded 140 mg/dL, and/or if the 2-hour value after oral glucose loading exceeded 200 mg/dL. Chronic infections and conditions known to be associated with recurrent episodes of infectious exacerbations were diagnosed after a detailed interview of the participant and a review of medical records.[15]

The same detailed procedure lead to the diagnosis of a history of cardiovascular diseases (composite of stroke, TIA, myocardial infarction, peripheral artery disease, definite angina and previous revascularization procedures). Myocardial infarction was deemed confirmed when World Health Organization criteria for definite disease status were met. Ischemic stroke and transient ischemic attacks were classified according to the criteria of the National Survey of Stroke. The diagnosis of symptomatic peripheral artery disease and angina pectoris required a positive response to the Rose questionnaire with the vascular nature of complaints confirmed by standard diagnostic procedures (ankle-brachial pressure index or angiography and exercise electrocardiogram or coronary angiography). Revascularization procedures (angioplasty and surgery) were carefully recorded. Ascertainment of events or procedures did not rely on hospital discharge codes or the patient's self-report but on a careful review of medical records provided by the general practitioners, death certificates, and Bruneck Hospital files, and the extensive clinical and laboratory examinations performed as part of the study protocols. Major advantages of the Bruneck Study are that virtually all subjects living in the Bruneck area were referred to the local hospital and that the network existing between the local hospital and the general practitioners allowed retrieval of practically all medical information on persons living in the area.

### Laboratory Methods

Blood samples were taken from the antecubital vein after subjects had fasted and abstained from smoking for >12 hours. CRP (hsCRP) was measured by immunonephelometry (Dade, Behring, Marburg, Germany) with a intra- and interassay coefficient of variation of 3.7%. Other standard lab parameters were measured by routine methods applied in the central lab facilities of Innsbruck University Clinics or Bruneck Hospital and have been described previously.[15]–[17]

PTX3 was measured by an in-house assay based on the murine monoclonal antibody MNB4 as capturing, and a rabbit antiserum (pAb) raised against human PTX3, affinity purified and biotinylated, as detection antibody. Streptavidin-horse radish peroxidase was used and absorbance at 450 mm (Abs_450_) was measured with an automatic ELISA reader. No cross reaction of MNB4 and pAb with human CRP and serum amyloid P component protein has been observed. For each biological sample, 2 dilutions in duplicate wells were evaluated and mean PTX3 content was calculated converting Abs_450_ values to protein concentration by means of a standard curve with recombinant purified human PTX3 (range from 75 pg/ml to 2.4 ng/ml). Detection limit of our assay is 100 pg/ml and the inter-assay variability ranges from 8 to 10%.[9]

Plasma G-CSF, SDF1-α, VEGF and MMP-9 levels were determined using commercially available kits (Quantikine, R&D Systems, UK) with the following intra/interassay coefficients of variation: G-CSF 3.6–6.2%/6.3–8.2%, SDF1-α 3.4–3.9%/8.2–13.4%, VEGF 4.5–6.7%/6.2–8.8% and MMP-9 1.9–2.9%/6.9–7.9%. All ELISA tests were carried out at room temperature on freshly thawed plasma samples. The concentration of all cytokines was determined by comparison with a standard curve, following manufacturer's instruction.

### Measurement of atherosclerosis

All participants underwent high-resolution B-mode ultrasonography with a 10 MHz imaging probe (ATL8 in the Bruneck and HDI3000 in the ARMY Study, both ATL Ultrasound, Bothell, Washington and General Electric Logic 7 in the ARFY Study, Milwaukee, Wisconsin). The scanning protocol involved the common carotid artery (CCA), the carotid bulb and internal carotid artery (ICA) at both sides. In addition femoral arteries were visualized in the Bruneck and the ARMY Study. All scans were using the same protocol with different scanning angles (anterior and posterolateral) employed to identify the greatest wall and plaque thickness and all measurements were performed by one and the same experienced sonographer (J.W.). The intima-media thickness (IMT) was assessed at the far wall as the distance between the interface of the lumen and intima, and the interface between the media and adventitia (Bruneck Study: intra-observer coefficient of variation, 7.9 percent (n = 100)). The maximal IMT was recorded and averaged for the left and right sides of the CCA (30 mm proximal to the carotid bulb), the carotid bulb, the ICA and the femoral artery (40 mm proximal to 10 mm distal of the bifurcation into the superficial and deep branches). As no normal values exist for young individuals “high IMT” in the ARFY and ARMY studies was assumed when the IMT value exceeded the 90^th^ percentile of IMT distribution in one or more of the segments explored. In the Bruneck Study, atherosclerotic lesions were defined by 2 ultrasound criteria: [1] wall surface (protrusion into the lumen) and [2] wall texture (echogenicity). The maximum axial diameter of plaques was assessed in the following vessel segments on the near and far wall of either side: proximal CCA (15 to 30 mm proximal to the carotid bulb), distal CCA (<15 proximal to the carotid bulb), proximal ICA (carotid bulb and initial 10 mm of the ICA), distal ICA (>10 mm above the flow divider), femoral artery (40 mm proximal to 10 mm distal of the bifurcation into the superficial and deep branches). The Atherosclerosis Score (AS) was calculated by addition of all plaque diameters of the carotid arteries (16 sites, intra-observer coefficient of variation, 13.5 percent (n = 100)) and the femoral arteries (2 sites). Ultrasound methodology has been extensively described previously.[14], [16], [17]

### Statistical Analysis

For statistical analysis the SPSS software package (version 18.0) was used. Continuous variables were presented as means or medians [inter-quartile range] and dichotomous variables as numbers (percentages). Participants were divided into three approximately equally sized groups according to tertiles of PTX3 (or hsCRP). Comparison of variable levels across these groups was performed using linear regression and logistic regression analysis of the variable of interest on age, sex and log_e_-transformed PTX3 or hsCRP level (P value for trend). Base models were adjusted for age and sex, multivariable models by age, sex, diabetes, hypertension, HDL and LDL cholesterol, smoking, BMI and waist circumference. To estimate the discriminative value of prediction models, i.e. the ability to correctly classify subjects into one of two categories, we calculated the C statistic, which is analogous to the area under the receiver-operating-characteristic curve (ROC) (larger values indicate better discrimination). Comparison of ROCs based on models including and not including PTX3 or hsCRP was performed according to the method of DeLong.[19] To assess model calibration or how closely the predicted probabilities reflect actual risk, we computed the Hosmer-Lemeshow calibration statistics comparing observed and predicted risk in decile categories of predicted risk (higher P values indicate better calibration). Not normally distributed variables were log-transformed (PTX3 and hsCPR). *P-values*≤0.05 were considered significant. When appropriate, Bonferroni adjustment of P values was applied.

## Results

**Figure 1:**
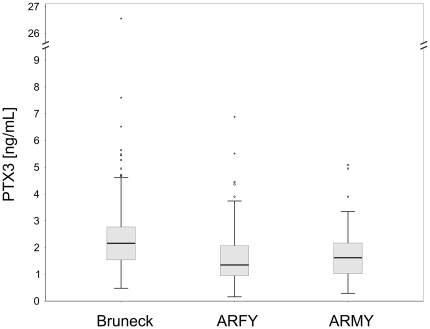
Distribution of PTX3 levels in the Bruneck (black), ARMY (blue) and ARFY (pink) study.

Median (IQR) PTX3 levels amounted 2.13 ng/mL (1.55–2.73) in the Bruneck (n = 562), 1.62 ng/mL (1.01–2.18) in the ARMY (n = 132) and 1.37 ng/mL (0.94–2.13) in the ARFY Study (n = 207). Distributions of PTX3 levels are depicted in Figure 1. In the Bruneck study representing a middle-aged and elderly male and female population, no differences in age and sex were observed across PTX3 tertile groups (Table 1). After adjustment for multiple testing, significant age- and sex-adjusted associations were found for neutrophil count and matrix-metalloproteinase 9 (MMP-9) level however not for chronic infection and pro-inflammatory vascular risk conditions (Table 1). The same analysis revealed numerous associations for hsCRP (Table 2).

**Table 1:** Population characteristics according to PTX3 tertile groups in the Bruneck Study.

	Tertile of PTX3 serum level [ng/mL]^a^			P value
	Low	Medium	High	PTX3^b^
**PTX3 (ng/mL)**				
mean±SD	1.32±0.30	2.11±0.20	3.43±1.87	
Range	0.48–1.75	1.76–2.46	2.47–26.52	
**Demographic variables**				
Age – years	67.16±8.7	68.62±9.747	71.92±9.76	0.054
Men – n (%)	83 (44.4)	94(50)	82(43.9)	0.607
**Lifestyle factors**				
Waist circumference – cm	91.92±11.22	92.36±11.76	89.78±13.20	0.041
Body mass index – kg/m^2^	26.40±3.84	26.20±4.00	25.31±4.50	0.016
Physical activity – score	2.46±0.72	2.43±0.75	2.29±0.63	0.042
Alcohol consumption – g/day	14.98±20.64	20.65±28.20	18.55±26.11	0.154
Smoking – pack-years	10.88±15.46	11.76±15.95	14.31±18.98	0.180
**Vascular risk factors**				
Diabetes mellitus – n (%)	14(7.5)	18(9.6)	26 (13.9)	0.100
Hypertension – n (%)	115 (61.5)	132 (70.2)	135 (72.2)	0.248
Fasting glucose – mg/dl	101.20±17.36	102.52±18.30	105.12±29.58	0.036
HbA1c – %	5.67±0.54	5.75±0.72	5.82±0.81	0.423
HDL-Cholesterol – mg/dL	63.63±13.24	64.17±14.82	64.34±13.86	0.436
LDL- Cholesterol – mg/dL	138.33±32.10	137.11±34.61	131.83±36.27	0.942
Triglycericdes ^d^ – mg/dL	119(94–165)	113(90–152)	114(91–155)	0.114
Lipoprotein(a) ^d^ – mg/dL	0.14(0.05–0.33)	0.15(0.05–0.47)	0.15(0.59–0.45)	0.512
Urinary ACR ^d^ – mg/g	6.2(3.6–14.5)	5.9(3.73–10.93)	8.9(4.6–23.1)	0.002
Fibrinogen ^d^ – mg/dL	286(254–318)	290(258–319)	301(271–352)	0.018
**Inflammatory Parameters**				
Chronic infection – n(%)	39 (20.9)	44 (23.4)	64 (34.2)	0.207
hsCRP– mg/dL	0.17(0.11–0.39)	0.18(0.10–0.39)	0.23(0.12–0.67)	0.004
Neutrophil count –/L	3277(2582–3930)	3386(2681–4436)	3752(3070–4787)	**<0.001**
MMP-9 – ng/mL	50.1(27.4–83.9)	66.3(33.1–92.0)	92.4(50.5–92.4)	**<0.001**
G-CSF – pg/mL	8.77(3.53–15.54)	9.57(5.23–15.53)	9.67(4.86–21.10)	0.402
SDF-1 – pg/mL	2486(2261–2823)	2501(2226–2806)	2620(2313–2974)	0.270
VEGF – pg/mL	61.4(24.1–140.6)	73.9(18.6–139.8)	91.9(46.2–169.8)	0.302

a Values presented are means±SD, medians (IQR) or numbers (%).

b P values are for trend and derived from age- and sex-adjusted linear or logistic regression analysis of each variable on log_e_-transformed PTX3 level. When correcting for multiple testing, a P value<0.002 **(in bold)** indicates statistical significance (Bonferroni adjustment).

Urinary ACR – Urinary albumin-to-creatinine ratio; hsCRP – high-sensitivity C-reactive Protein; MMP-9 – matrix metalloproteinase-9; G-CSF – granulocyte colony stimulating factor; SDF-1 – stromal cell-derived factor-1; VEGF – vascular endothelial growth factor.

**Table 2:** Population characteristics according to CRP tertile groups in the Bruneck Study.

	Tertile of CRP serum level [mg/L]^a^			P value
	Low	Medium	High	hsCRP^b^
**CRP (mg/L)**				
Mean±SD	0.88±0.34	2.09±0.45	9.24±1.07	
Range	0.02–1.4	1.5–3.2	3.3–75.40	
**Demographic variables**				
Age – years	67.8±9.3	68.9±9.5	71.0±9.8	**<0.001**
Men – n (%)	95 (48.2)	78 (45.1)	85 (44.7)	0.568
**Lifestyle factors**				
Waist circumference – cm	88.1±11.6	90.7±11.8	95.3±11.8	**<0.001**
Body mass index – kg/m^2^	24.9±3.8	25.9±3.7	27.2±4.5	**<0.001**
Physical activity – score	2.46±0.72	2.43±0.68	2.27±0.70	0.036
Alcohol consumption – g/day	19.7±27.8	16.9±23.9	17.5±23.7	0.365
Smoking – pack-years	11.2±14.6	9.9±14.9	15.4±20.1	**0.001**
**Vascular risk factors**				
Diabetes mellitus – n (%)	15 (7.6)	15 (8.6)	28 (14.7)	0.016
Hypertension – n (%)	115 (58.4)	118 (67.4)	149 (78.4)	**<0.001**
Fasting glucose – mg/dl	101.5±24.7	101.5±16.5	105.8±24.5	0.071
HbA1c – %	5.66±0.63	5.73±0.54	5.86±0.88	0.016
HDL-Cholesterol – mg/dL	66.9±14.2	63.9±13.5	61.2±13.6	**<0.001**
LDL- Cholesterol – mg/dL	136.1±33.8	135.86±32.33	135.87±37.04	0.937

**Figure 2:**
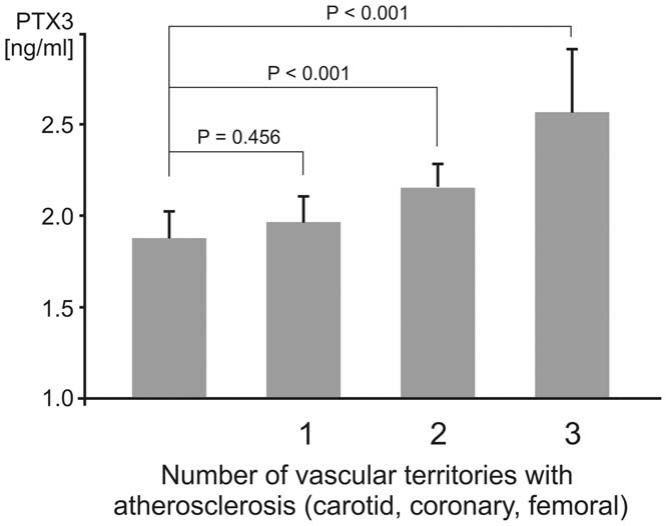
PTX3 level according to the number of vascular territories involved in the atherosclerosis process (Bruneck Study). Presence of atherosclerosis was determined by high-resolution ultrasound in the (common and internal) carotid (one territory) and femoral arteries (one territory), and on clinical grounds in the coronary arteries (clinically overt CHD, one territory). PTX3 levels were expressed as age and sex adjusted geometric means. Whiskers represent the 95% confidence interval (CI). P value for trend <0.001 (analysis adjusted for sex and age) and P = 0.003 (analysis adjusted sex, age, HDL and LDL-cholesterol, diabetes, smoking, hypertension, body mass index and waist circumference). Post hoc P values for comparisons between groups were calculated by the Dunnett T-Test.

**Figure 3:**
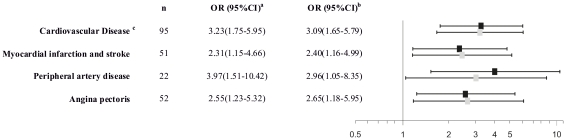
Logistic regression analysis of PTX3 level on prevalent vascular diseases. Odds ratios (OR) and 95% confidence intervals (CI) were calculated per 1 unit increase in log-transformed PTX3. Black squares denote ORs adjusted for sex and age, gray squares mark ORs adjusted for age, sex, HDL, LDL-cholesterol, smoking, diabetes, hypertension, body mass index and waist circumference. ^a^ adjusted for age and sex. ^b^ adjusted for age, sex, HDL, LDL-cholesterol, smoking, diabetes, hypertension, body mass index and waist circumference. ^c^ composite endpoint of stroke, TIA, myocardial infarction, peripheral artery disease, definite angina and previous revascularization procedures.

Next, we analyzed for a potential association between IMT - a measure of early vessel pathology - and PTX3 level. In the Bruneck Study, PTX3 level emerged as unrelated to the mean maximum IMT assessed at the common carotid, internal carotid and femoral artery (Table 3). In line, median [interquartilerange] PTX3 level did not differ between healthy young individuals with or without a high IMT in the ARMY (1.63 [0.59–2.67] vs. 1.64 [0.40–2.88] ng/mL, P = 0.734) and ARFY Study (1.37 [0.68–2.07] vs. 1.37 [0.73–2.01] ng/mL, P = 0.785). In contrast, severity (atherosclerosis score) of carotid and femoral atherosclerosis was found to be independently and significantly associated with PTX3 level in the Bruneck Study (Table 2). The corresponding results for hsCRP are summarized in Table 4. In addition, PTX3 level increased with the number of vascular beds involved in the atherosclerosis process (P<0.001, Figure 2). The finding extends to manifest cardiovascular disease (composite endpoint) and its individual disease components angina, peripheral artery disease, myocardial infarction and stroke (Figure 3). Addition of PTX3 to a model including standard vascular risk factors improved its predictive accuracy for cardiovascular disease (Table 5) and the overall performance was better than that observed for hsCRP (Table 5).

**Table 3:** Measures of carotid and femoral artery atherosclerosis according to PTX3 tertile groups in the Bruneck Study.

	Tertile of PTX3 serum level			P value	
	Low	Medium	High	All	No CVD
Common carotid artery IMT – mm	1.00±0.20	0.99±0.19	1.05±0.22	0.729 (0.940)	0.923 (0.983)
Internal carotid artery IMT – mm	0.86±0.15	087±0.18	0.90±0.17	0.455 (0.435)	0.823 (0.844)
Femoral artery IMT – mm	0.95±0.21	0.99±0.20	1.03±0.21	0.097 (0.174)	0.113 (0.168)
Carotid artery AS – mm	4.11±5.73	4.51±5.17	6.67±7.23	**0.002 (0.013)**	0.398 (0.521)
Femoral artery AS – mm	1.96±2.41	2.45±2.62	3.16±2.91	**0.001 (0.005)**	**0.003 (0.009)**

Values presented are means±SD. Analyses were conducted in the entire population (All) and separately in those free of CVD (No CVD). P values are for trend and derived from age/sex-adjusted and multivariable (adjustment for age, sex, diabetes, hypertension, HDL and LDL cholesterol, smoking body mass index and waist circumference) linear regression analysis of each variable on log_e_-transformed PTX3 level. P values in brackets are those from the multivariable models. AS, atherosclerosis score.

**Table 4:** Measures of carotid and femoral artery atherosclerosis according to CRP tertile groups in the Bruneck Study.

	Tertile of CRP serum level			P value	
	Low	Medium	High	All	No CVD
Common carotid artery IMT – mm	0.98±0.18	1.00±0.20	1.07±0.22	**<0.001** (0.052)	**0.010** (0.154)
Internal carotid artery IMT – mm	0.86±0.15	0.86±0.17	0.91±0.16	0.072 (0.410)	0.391 (0.824)
Femoral artery IMT – mm	0.95±0.20	0.98±0.21	1.04±0.19	**<0.001 (0.006)**	**<0.001 (0.018)**
Carotid artery AS – mm	4.06±5.73	4.54±5.49	6.67±6.97	**0.001 (0.007)**	**0.031** (0.118)
Femoral artery AS – mm	2.40±2.61	2.19±2.53	2.96±2.88	0.187 (0.621)	0.167 (0.431)

Values presented are means±SD. Analyses were conducted in the entire population (All) and separately in those free of CVD (No CVD). P values are for trend and derived from age/sex-adjusted and multivariable (adjustment see Table 3) linear regression analysis of each variable on log_e_-transformed PTX3 level. P values in brackets are those from the multivariable models. AS, atherosclerosis score.

**Table 5:** Contribution of PTX3 and C-reactive protein level or the combination of the two to the risk prediction of prevalent cardiovascular disease.

Model/Variables	Odds ratio (95%CI) per 1-SD unit increase in variable level	Variable LR Chi-Square†	P value†	Calibration P Value‡ Hosmer-Lemeshow calibration statistics	Model Discrimination C-Statistic§ (95%CI)
**Multivariable model** *		406.11	<0.001	0.679	0.811 (0.765–0.858)
**+PTX3** #	1.64 (1.25–2.17)	13.44	<0.001	0.903	0.822 (0.776–0.869)
**+hsCRP** #	1.29 (1.00–1.67)	3.91	0.050	0.407	0.814 (0.769–0.860)
**+PTX3** #	1.60 (1.21–2.11)	13.44	<0.001		
**+hsCRP** #	1.21 (0.93–1.56)	2.03	0.154	0.915	0.825 (0.779–0.871)

*The multivariable logistic regression model was adjusted c. The composite cardiovascular endpoint subsumes all cases of ischemic stroke, TIA, myocardial infarction, peripheral artery disease, definite angina and previous revascularization procedures (n = 95).

† Variable chi-square is the 1-degree-of-freedom likelihood ratio chi-square statistic with P values for inclusion of each variable separately to the multivariable base model, with larger values indicating greater improvement in fit. The+sign indicates the addition of either PTX3 level, or C-reactive protein level, or both to the base model considering standard risk factors.

‡ The calibration P values was calculated with the Hosmer-Lemeshow calibration statistics comparing observed and predicted risk in decile categories of predicted risk. Higher values for the calibration P value reflect better fit.

§ The C statistic represents the area under the receiver-operating-characteristic curve. Higher values for the C statistic reflect better fit.

# non normally distributed PTX3 and hsCRP levels were log-transformed.

## Discussion

In the large and well-defined population of the Bruneck Study, PTX3 level showed little associations with classic and pro-inflammatory risk parameters which contrasts to hsCRP taking part in acute phase response and being up-regulated under a variety of inflammatory conditions (Table 2 and [20], [21]). As to PTX3, only two correlations with neutrophil count and MMP-9 concentration remained significant after correction for multiple testing. MMP-9 like PTX3 is primarily expressed in bone marrow-derived cells in the atherosclerotic plaque, plays a role in the modulation of local inflammation and contributes to plaque destabilization.[22] High serum levels of MMP-9 have previously been linked to femoral atherosclerosis and cardiovascular disease.[22], [23] The robust association between PTX3 level and neutrophil count fits well to the observation that almost all neutrophiles, infiltrating the atherosclerotic plaques and present in coronary arterial thrombi, express PTX3.[24]

So far, PTX3 level has been proposed as a risk predictor for acute myocardial infarction [5] and biomarker of adverse outcome in patients with unstable angina pectoris [25], myocardial infarction [9] and heart failure [10]. Smaller evaluations in patients with chronic kidney disease demonstrate an association between PTX3 levels and prevalent [26]–[28] as well as future [29] CVD. In a subgroup of the Cardiovascular Health Study high baseline PTX3 levels were linked with the subsequent risk of cardiovascular and all cause death.[11] In line with the latter, as well as two very recent publications [29], [30], PTX3 levels were not associated with early stage atherosclerosis (IMT) in the Bruneck, ARMY and ARFY studies (Table 3). To the best of our knowledge this is the first study to describe a significant and independent association of PTX3 with advanced human atherosclerosis (Table 3). Our findings fit particularly well to murine and human *ex vivo* data suggesting pronounced PTX3 expression in advanced but not early atherosclerotic lesions.[12], [13] In the Bruneck Study, PTX3 was independently related to both carotid and femoral atherosclerosis and to manifest cardiovascular diseases mainly originating from unstable plaques (Figure 3). Moreover, PTX3 level increased with the number of vascular beds involved in the atherosclerotic process (Figure 2). Finally, preliminary data suggest PTX3 level to be superior to hsCRP in predicting prevalent cardiovascular disease (Table 5). However, these data have to be viewed in light of the relatively small number of participants (n = 562), limited number of outcome events and lack of serial measurements of PTX3 and IMT in given individuals so far. While the Bruneck study is representative of the general community the ARFY study enrolled students of different healthcare professions and thus a selected sample.

Our epidemiological study cannot establish whether the association between high PTX levels and advanced atherosclerosis reflects causal involvement, an epiphenomenon or counter-regulatory protective mechanism. Experimental data support the latter view. PTX3 knock-out mice with an atherosclerosis-prone apoE^−/−^ background were shown to develop more pronounced atherosclerosis than those expressing PTX3 with the extra atherosclerosis being reversed by administration of PTX3.[13] More experimental and prospective epidemiological studies are needed to further establish the mechanistic role of PTX3 in human atherosclerosis.

### Conclusion

In summary, PTX3 serum level is a marker of advanced and symptomatic but not early atherosclerosis in humans. When compared to CRP, PTX3 predicted prevalent cardiovascular disease better, had fewer associations with other vascular risk conditions and may be more specific for vascular wall inflammation.

## References

[1] Ross R (1999). Atherosclerosis–an inflammatory disease.. N Engl J Med.

[2] Hansson GK, Libby P, Schonbeck U, Yan ZQ (2002). Innate and adaptive immunity in the pathogenesis of atherosclerosis.. Circ Res.

[3] Wick G, Knoflach M, Xu Q (2004). Autoimmune and Inflammatory Mechanisms in Atherosclerosis.. Annu Rev Immunol.

[4] Bottazzi B, Doni A, Garlanda C, Mantovani A (2010). An integrated view of humoral innate immunity: pentraxins as a paradigm.. Annu Rev Immunol.

[5] Peri G, Introna M, Corradi D, Iacuitti G, Signorini S (2000). PTX3, A prototypical long pentraxin, is an early indicator of acute myocardial infarction in humans.. Circulation.

[6] Mantovani A, Garlanda C, Bottazzi B, Peri G, Doni A (2006). The long pentraxin PTX3 in vascular pathology.. Vascul Pharmacol.

[7] Kaess BM, Vasan RS (2011). Heart failure: Pentraxin 3-a marker of diastolic dysfunction and HF?. Nat Rev Cardiol.

[8] Inoue K, Sugiyama A, Reid PC, Ito Y, Miyauchi K (2007). Establishment of a high sensitivity plasma assay for human pentraxin3 as a marker for unstable angina pectoris.. Arterioscler Thromb Vasc Biol.

[9] Latini R, Maggioni AP, Peri G, Gonzini L, Lucci D (2004). Prognostic significance of the long pentraxin PTX3 in acute myocardial infarction.. Circulation.

[10] Suzuki S, Takeishi Y, Niizeki T, Koyama Y, Kitahara T (2008). Pentraxin 3, a new marker for vascular inflammation, predicts adverse clinical outcomes in patients with heart failure.. Am Heart J.

[11] Jenny NS, Arnold AM, Kuller LH, Tracy RP, Psaty BM (2009). Associations of pentraxin 3 with cardiovascular disease and all-cause death: the Cardiovascular Health Study.. Arterioscler Thromb Vasc Biol.

[12] Rolph MS, Zimmer S, Bottazzi B, Garlanda C, Mantovani A (2002). Production of the long pentraxin PTX3 in advanced atherosclerotic plaques.. Arterioscler Thromb Vasc Biol.

[13] Norata GD, Marchesi P, Pulakazhi Venu VK, Pasqualini F, Anselmo A (2009). Deficiency of the long pentraxin PTX3 promotes vascular inflammation and atherosclerosis.. Circulation.

[14] Kiechl S, Willeit J (1999). The natural course of atherosclerosis.. Arterioscler Thromb Vasc Biol.

[15] Kiechl S, Egger G, Mayr M, Wiedermann CJ, Bonora E (2001). Chronic infections and the risk of carotid atherosclerosis: prospective results from a large population study.. Circulation.

[16] Knoflach M, Kiechl S, Kind M, Said M, Sief R (2003). Cardiovascular risk factors and atherosclerosis in young males: ARMY study (Atherosclerosis Risk-Factors in Male Youngsters).. Circulation.

[17] Knoflach M, Kiechl S, Penz D, Zangerle A, Schmidauer C (2009). Cardiovascular risk factors and atherosclerosis in young women: atherosclerosis risk factors in female youngsters (ARFY study).. Stroke.

[18] Baecke JA, Burema J, Frijters JE (1982). A short questionnaire for the measurement of habitual physical activity in epidemiological studies.. Am J Clin Nutr.

[19] DeLong ER, DeLong DM, Clarke-Pearson DL (1988). Comparing the areas under two or more correlated receiver operating characteristic curves: a nonparametric approach.. Biometrics.

[20] Rohde LE, Hennekens CH, Ridker PM (1999). Survey of C-reactive protein and cardiovascular risk factors in apparently healthy men.. Am J Cardiol.

[21] Yudkin JS, Kumari M, Humphries SE, Mohamed-Ali V (2000). Inflammation, obesity, stress and coronary heart disease: is interleukin-6 the link?. Atherosclerosis.

[22] Back M, Ketelhuth DF, Agewall S (2010). Matrix metalloproteinases in atherothrombosis.. Prog Cardiovasc Dis.

[23] Olson FJ, Schmidt C, Gummesson A, Sigurdardottir V, Hulthe J (2008). Circulating matrix metalloproteinase 9 levels in relation to sampling methods, femoral and carotid atherosclerosis.. J Intern Med.

[24] Savchenko A, Imamura M, Ohashi R, Jiang S, Kawasaki T (2008). Expression of pentraxin 3 (PTX3) in human atherosclerotic lesions.. J Pathol.

[25] Matsui S, Ishii J, Kitagawa F, Kuno A, Hattori K (2010). Pentraxin 3 in unstable angina and non-ST-segment elevation myocardial infarction.. Atherosclerosis.

[26] Nishi K, Imamura T, Kitamura K, Ogawa T, Fujimoto S (2011). Associations of plasma pentraxin 3 and monocyte chemoattractant protein-1 concentrations with cardiovascular disease in patients with chronic kidney disease.. Ren Fail.

[27] Xu Y, Ding X, Zou J, Liu Z, Jiang S (2011). Plasma Pentraxin 3 is Associated with Cardiovascular Disease in Hemodialysis Patients.. Ren Fail.

[28] Kanbay M, Ikizek M, Solak Y, Selcoki Y, Uysal S (2011). Uric acid and pentraxin-3 levels are independently associated with coronary artery disease risk in patients with stage 2 and 3 kidney disease.. Am J Nephrol.

[29] Yilmaz MI, Sonmez A, Ortiz A, Saglam M, Kilic S (2011). Soluble TWEAK and PTX3 in nondialysis CKD patients: impact on endothelial dysfunction and cardiovascular outcomes.. Clin J Am Soc Nephrol.

[30] Jylhava J, Haarala A, Kahonen M, Lehtimaki T, Jula A (2011). Pentraxin 3 (PTX3) is associated with cardiovascular risk factors: the Health 2000 Survey.. Clin Exp Immunol.

